# Smad4 and Eomes converge on a shared transcriptional program associated with inflammatory CD8 T-cell differentiation

**DOI:** 10.3389/fimmu.2026.1858529

**Published:** 2026-08-17

**Authors:** Karthik Chandiran, Nandu C. Karingari, Srineeharika Sirigineedi, Linda S. Cauley

**Affiliations:** 1 School of Biology, Indian Institute of Science Education and Research, Thiruvananthapuram, India; 2 Department of Immunology, UConn Health, Farmington, CT, United States

**Keywords:** CD8 T cells, ChIPseq, Eomes: Eomesodermin, RNAseq, Smad3, Smad4, TGF-beta signaling

## Abstract

The ability of CD8 T cells to mediate protective immunity while limiting immunopathology depends on critical transcription factors such as Smad4 and Eomes that regulate differentiation of activated CTLs. Disruption of transcriptional programs mediated by Eomes and Smad4 in activated CTLs has been implicated in viral infections, chronic inflammation and cancer. Smad4 and Eomes have been shown to be critical for differentiation of memory cells, particularly TRM cells. To understand the gene regulation programs mediated by Smad4 and Eomes, we have done an integrated RNAseq and ChIPseq analysis using CD8 T cells that are isolated from Smad4KO (S4KO), EomesKO, and Smad3/Smad4-DKO (S34-DKO) mice. We identified overlapping and distinct gene expression programs mediated by Smad4 and Eomes that are associated with shaping CD8 T cell identity and functional bias. They act as critical regulators of genes involved in T cell differentiation, metabolic programming and epigenetic control in CD8 T cells. Smad4 and Eomes regulate the genes involved in tissue residency and OXPHOS-linked mitochondrial bioenergetics. Finally, we showed that canonical TGF-beta signaling is active in the absence of Smad4 or Eomes and abrogated only when both Smad3 and Smad4 are deleted. Surprisingly, CD8 T cells lacking both Smad3 and Smad4 are more enriched for TRM signature genes compared to Smad4 or Eomes-deficient CD8 T cells.

## Introduction

The functionality of T cells is largely determined by their being present at the right place at the right time, especially during viral infections. During acute respiratory viral infection, including those caused by influenza and coronaviruses, CD8 T cell-mediated immunity acts as a double-edged sword. Appropriately differentiated effector T cells are required for mounting an effective immune response; however, aberrant differentiation of activated cytotoxic T lymphocytes (CTLs) due to their environmental niche could be detrimental to the host. A wide variety of factors can be attributed to the differentiation and localization of activated CTLs, such as costimulatory molecules (1), cytokines (2–5), transcription factors including T-bet/Eomesodermin (Eomes) (6, 7), Zeb1/Zeb2 (8) and Blimp-1 (9), and even RNA-binding proteins in certain cases (10).

Transforming growth factor-beta (TGFβ) is a pleiotropic cytokine that belongs to the TGFβ superfamily comprising TGFβ (TGFβ1–3), bone morphogenic proteins (BMPs), growth differentiation factors (GDFs), activins, inhibins, and nodal. The canonical signaling is mediated by Small (Sma) and mothers against decapentaplegic (Mad)-related (SMAD) proteins (11) whereas non-canonical signaling is mediated through SMAD-independent mechanisms (12, 13). Depending on the ligand and the receptor activated, different receptor-SMADs (R-SMAD: Smad-1,5, and 8 or Smad-2 and 3) associate with the common mediator (Smad4) to form transcriptionally active trimeric complexes that translocate to the nucleus and regulate gene expression (14, 15). In some instances, SMAD4 is not required and has been replaced by another R-SMAD in the trimer for gene regulation (16, 17).

TGFβ signaling has been widely shown to influence the fate of CD8 T cells by suppressing proliferation and cytotoxicity while inducing memory cell differentiation and tissue residency (18–20). In addition, TGFβ can induce the expression of CD103 and precondition naïve CD8 T cells to become resident memory cells (T_RM_) with the help of migratory DCs (21). Paradoxically, the intracellular signaling component Smad4 does the exact opposite, where it induces the formation of KLRG1+ effector cells (T_EFF_) and inhibits the formation of T_RM_ cells (22–24). Supporting this observation, multiple groups have reported that Smad4 can act independently of TGFβ during differentiation of T_RM_ (24–27). We have previously reported that Smad4 exerts its effect on CD8 T cells directly by inhibiting molecules such as CD103 that facilitate residency and also through inducing the expression of Eomes, another key transcription factor in T cell differentiation (24).

Although Eomes and Smad4 deficiency do not affect the development, homeostasis, and activation of naïve T cells or cytolytic ability of activated CTLs, they have a significant effect on the differentiation of activated CTLs (22, 23, 28). Eomes, a T-box transcription factor, synergizes with TGFβ/Smad4 to drive memory precursor formation (5, 29). However, Eomes, similar to Smad4, suppresses the formation of T_RM_ cells by inhibiting the expression of *itgae*, the gene that codes for CD103 (5, 24). Eomes and Smad4 operate independently of each other in regulating memory cell differentiation because the absence of both Eomes and Smad4 has an additive effect on the expression of CD103 during influenza infection (24).

The ability of CD8 T cells to mediate protective immunity while limiting immunopathology depends on critical transcription factors, such as Smad4 and Eomes, that regulate differentiation of activated CTLs. Disruption of transcriptional programs mediated by Eomes (30–32) and Smad4 (26, 33, 34) in activated CTLs has been implicated in viral infections, chronic inflammation, and cancer. Smad4 and Eomes have been shown to be critical for differentiation of memory cells, particularly TRM cells. To understand the gene regulation programs mediated by Smad4 and Eomes, we have done an integrated RNAseq and ChIPseq analysis using CD8 T cells that are isolated from Smad4KO (S4KO), EomesKO, and Smad3/Smad4-DKO (S34-DKO) mice.

We identified overlapping and distinct gene expression programs associated with Smad4 and Eomes that shape activated CD8 T cell identity and functional bias. They act as critical regulators of genes involved in T cell differentiation, metabolic programming, and epigenetic control in CD8 T cells. Smad4 and Eomes regulate the genes involved in tissue-residency and OXPHOS-linked mitochondrial bioenergetics. Finally, we showed that canonical TGFβ signaling is active in the absence of Smad4 or Eomes and is abrogated only when both Smad3 and Smad4 are deleted. Surprisingly, CD8 T cells lacking both Smad3 and Smad4 are more enriched for TRM signature genes compared to Smad4 or Eomes-deficient CD8 T cells.

## Method

### Mice

Mice were housed at the University of Connecticut Health Center (UCONN Health) in accordance with institutional guidelines. Experiments were performed in accordance with protocols approved by the UCONN Health Institutional Animal Care and Use Committee (IACUC). Mice that express Cre recombinase under the control of the distal-Lck promoter (dLck) were used to generate conditional knockout mice by crossing them with Smad3^fl/fl^, Smad4^fl/fl^, and Eomes^fl/fl^. All the animals were purchased from The Jackson Laboratory and bred and maintained on a C57BL/6 background under specific-pathogen-free conditions. The experiments were performed using age-matched female mice between 8 and 12 weeks of age.

### Cell culture

Naïve CD8 T cells were isolated from spleen and lymph node using Mojosort isolation kits (Biolegend, Dedham, MA). For T cell activation, naïve CD8 T cells were stimulated with plate-bound anti-CD3, where each well in a 12-well plate is coated with 500ul anti-CD3 diluted in PBS at 4ug/ml. Naïve CD8 T cells are activated with anti-CD28 (1ug/ml) and rIL2 (20U/ml) in complete RPMI with 10% FBS, 2mM L-glutamine, 100 U/ml Penicillin-Streptomycin, 100mM HEPES, 1mM sodium pyruvate, and 50uM β-mercaptoethanol.

### RNA-seq and computational analysis

Total RNA was isolated using the RNeasy plus kit (Qiagen, #74134), and the quality was evaluated using the Bioanalyzer from Agilent technologies. The libraries were created using the Illumina TruSeq RNA sample prep kit (Illumina, San Diego, USA). RNA libraries were subjected to sequencing for paired-end 2x100bp. Reads were trimmed using sickle, and quality-controlled reads were aligned to mouse genome (mm39) using HISAT2. BAM file conversion, sorting, and indexing were done with Samtools. Read counts were obtained from resulting BAM files using htseq-count. For transcriptomic analysis, differentially expressed genes were identified using DESeq2 in R. The plots were visualized using the ggplot2 package, heatmaps were created with the pheatmap package, volcano plots were created using the EnhancedVolcano package, and the Venn diagrams were created using the eulerr package in R. KEGG and Gene Ontology (GO) analysis were done using clusterProfiler in R. The genes with an adjusted p-value < 0.05 and |log2foldchange| > 1 were considered significantly differentially expressed and used for pre-ranked GSEA (Broad Institute, San Diego, CA) and fgsea package in R. Differentially expressed genes were compared with previously published datasets using GEO accession# GSE39152 (35) and GSE70813 (36). Bulk RNA-seq data generated in this study are available at the National Center for Biotechnology Information (NCBI) Gene Expression Omnibus (GEO) under accession code GSE338265.

### ChIP-seq analysis

Raw reads from previously published datasets (Accession number: GSE118174, GSE192386) were used for analysis (37, 38). Reads were trimmed using sickle, and quality-controlled reads were aligned to the mouse genome (mm39) using bowtie2. BAM file conversion, sorting, indexing, and pcr duplicate removal were done with Samtools. ChIP-seq peaks were called for each sample versus a control sample using MACS3. Genes proximal to each peak (-1000 to 1000 bp from the transcriptional start site) were annotated with ChIPseeker using narrowPeaks obtained from peak calling (39).

## Results

### Smad4 deficiency induces widespread transcriptional and metabolic reprogramming in activated CD8 T cells

**Figure 1:**
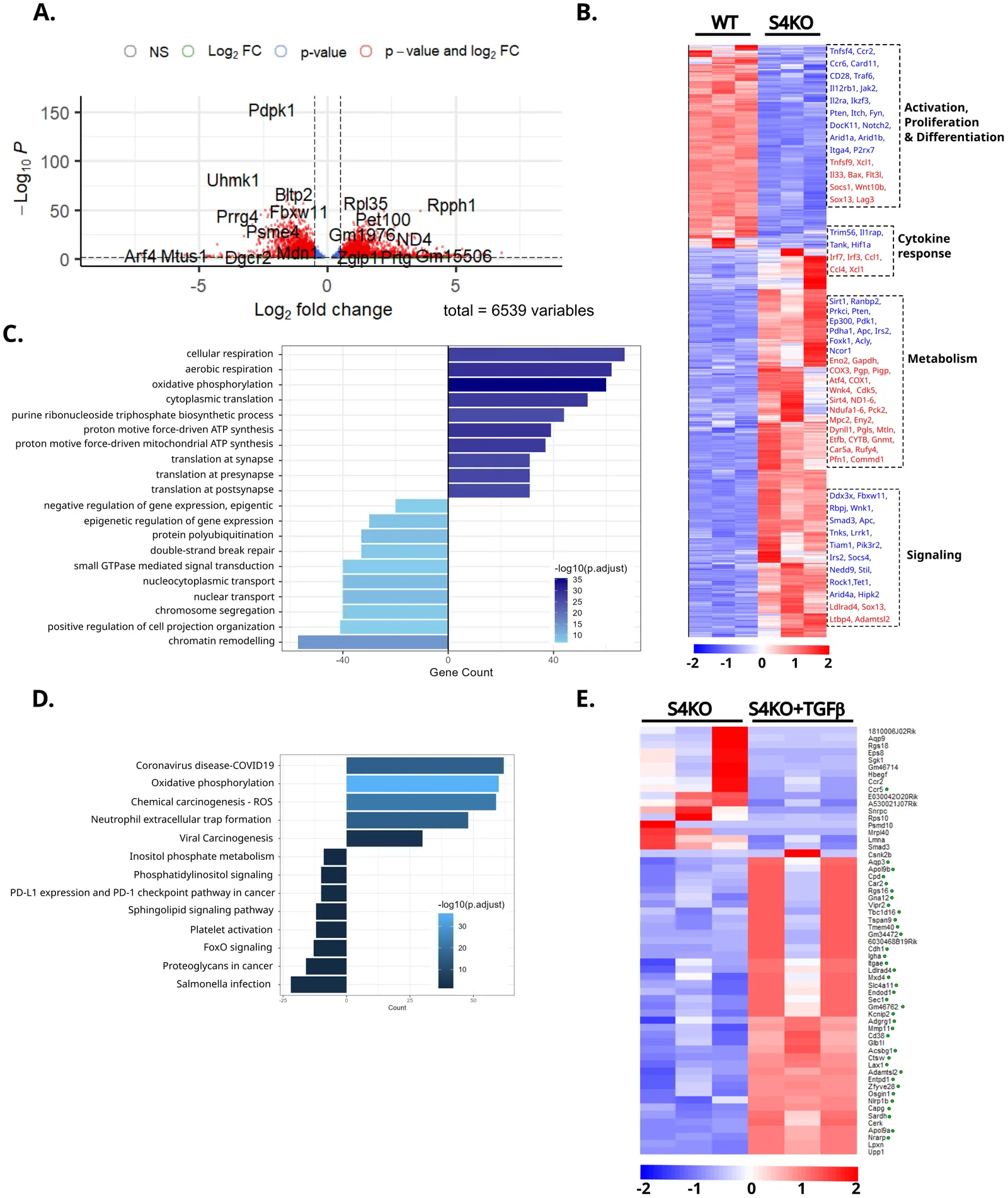
Smad4 deficiency induces transcriptional reprogramming of genes involved in differentiation and metabolism in activated CD8 T cells. **(A)** Volcano plot and **(B)** Heatmap of differentially expressed genes in S4KO CD8 T cells activated *in vitro* for 48 hours and expanded without TGFβ. **(C)** Gene Ontology and **(D)** KEGG analysis of differentially expressed genes. **(E)** Heatmap of differentially expressed genes in S4KO CD8 T cells when exposed to TGFβ (10ng/ml). The green dot highlights the genes that are differentially expressed in WT CD8 T cells. Each lane in the heatmaps represents CD8 T cells isolated from one animal (WT (n=3), S4KO (n=3) – 8–12 weeks old. All mice were females).

To decipher the role of Smad4 during T cell activation and differentiation, we activated CD8 T cells isolated from wild-type (WT) and Smad4-deficient (S4KO) mice *in vitro* for 48 hours and expanded them further for an additional 12 hours in the presence/absence of exogenous TGFβ to identify genes that are altered during initial stages of differentiation. These cells were subjected to unbiased bulk RNA sequencing (RNA-seq) post-activation and expansion. The reads mapped to the mouse genome (mm39) were analyzed using DESeq2 in R. In samples obtained immediately after 48 hours of activation, there were 3804 genes that were differentially expressed in S4KO, among which 589 genes showed 2-fold or greater expression (**Supplementary Figures 1A, B**, **Supplementary Table 1**). Based on Gene Ontology and KEGG analysis, the differentially expressed genes were mostly involved in metabolic pathways (**Supplementary Figures 1C, D**). In cells that were further expanded for an additional 12 hours, 6539 genes were differentially expressed (p_adj_ < 0.05) (**Figure 1A**), among which 2584 genes showed 2-fold or greater expression (**Figure 1B**, **Supplementary Table 2**). Smad4 deficiency induced attenuated expression of genes involved in TCR signaling and altered expression of co-stimulatory molecules. Here, the expression of *Cd28* and *Tnfsf4* (encodes OX40L) is decreased, and the expression of *Tnfsf9* is increased (encodes 4-1BBL) (**Figure 1B**). Smad4-deficient cells showed reduced expression of genes involved in proximal-TCR signaling (*Pik3r2, Irs2*, and *Nedd9*), Wnt/β-catenin signaling (*Apc, Tnks*, and *Rbpj*), cytoskeletal organization (*Rock1, Lrrk1*, and *Wnk1*), and chromatin remodeling (*Tet1, Arid1a, Arid1b, Arid4a*, and *Hipk2*) (**Figure 1B**). Smad4 deficiency also led to decreased expression of cytokine receptors (*Il2ra* and *Il12rb1*) and *Jak2*, which codes for the transcription factor facilitating JAK-STAT signaling in response to secreted cytokines (**Figure 1B**). Conversely, *Irf3* and *Irf7* were upregulated in S4KO cells, implying enhanced responsiveness to type-1 interferon signaling suggesting an altered response to cytokines (**Figure 1B**). Notably, Smad4 regulated chemokines and chemokine receptors involved in migration, homing, and recruitment of immune cells. In the absence of Smad4, we observed a decrease in *Ccr2* and *Ccr6* and an increase in *Ccl1*, *Ccl4*, and *Xcl1* (**Figure 1B**).

In addition to regulating components involved in TCR signaling and differentiation, Smad4 also strongly regulates the expression of genes involved in T cell metabolism. S4KO cells have decreased expression of key regulators of quiescence and oxidative control, including *Sirt1, Pten, Pdha, Apc, Foxk1, Ncor1*, and *Prkci*, while increasing *Sirt4* and mitochondrial respiratory components (*Cox1, Cox3, ND 1-6*, and *Ndufa 1-6*) (**Figure 1B**). The combined elevation of *Sirt4* and decrease of *Pdk1* and *Sirt1* suggests a shift from glycolytic gating towards enhanced pyruvate oxidation and mitochondrial respiration. Gene Ontology analysis revealed significant enrichment of genes involved in oxidative phosphorylation and mitochondrial respiration (**Figure 1C**). KEGG analysis indicated that in the absence of Smad4, genes involved in viral infection and carcinogenesis are overexpressed, whereas genes involved in PD-1 checkpoint blockade and bacterial infection are reduced (**Figure 1D**).

Interestingly, the TGFβ signaling pathway was still found to be active in Smad4-deficient cells, albeit in a limited capacity. Activated WT cells expanded in the presence of TGFβ (10ng/ml) resulted in differential expression of 590 genes (padj <0.05), among which 231 genes showed 2-fold or greater expression (**Supplementary Table 3**). However, S4KO cells still caused differential expression in 161 genes (padj<0.05), with 59 genes showing 2-fold or greater in the presence of exogenous TGFβ (**Figure 1E**, **Supplementary Table 4**). Comparison of genes showing 2-fold or greater expression between WT and S4KO revealed that more than two-thirds of the genes in S4KO are similarly regulated by TGFβ in WT (**Figure 1E**). These findings altogether suggest that Smad4 regulates different aspects of T cell differentiation and the absence of Smad4 generates a dysregulated and dysfunctional T cell with intact TGFβ signaling.

### Loss of Eomes drives a transcriptional program similar to Smad4-deficient CD8 T cells

**Figure 2:**
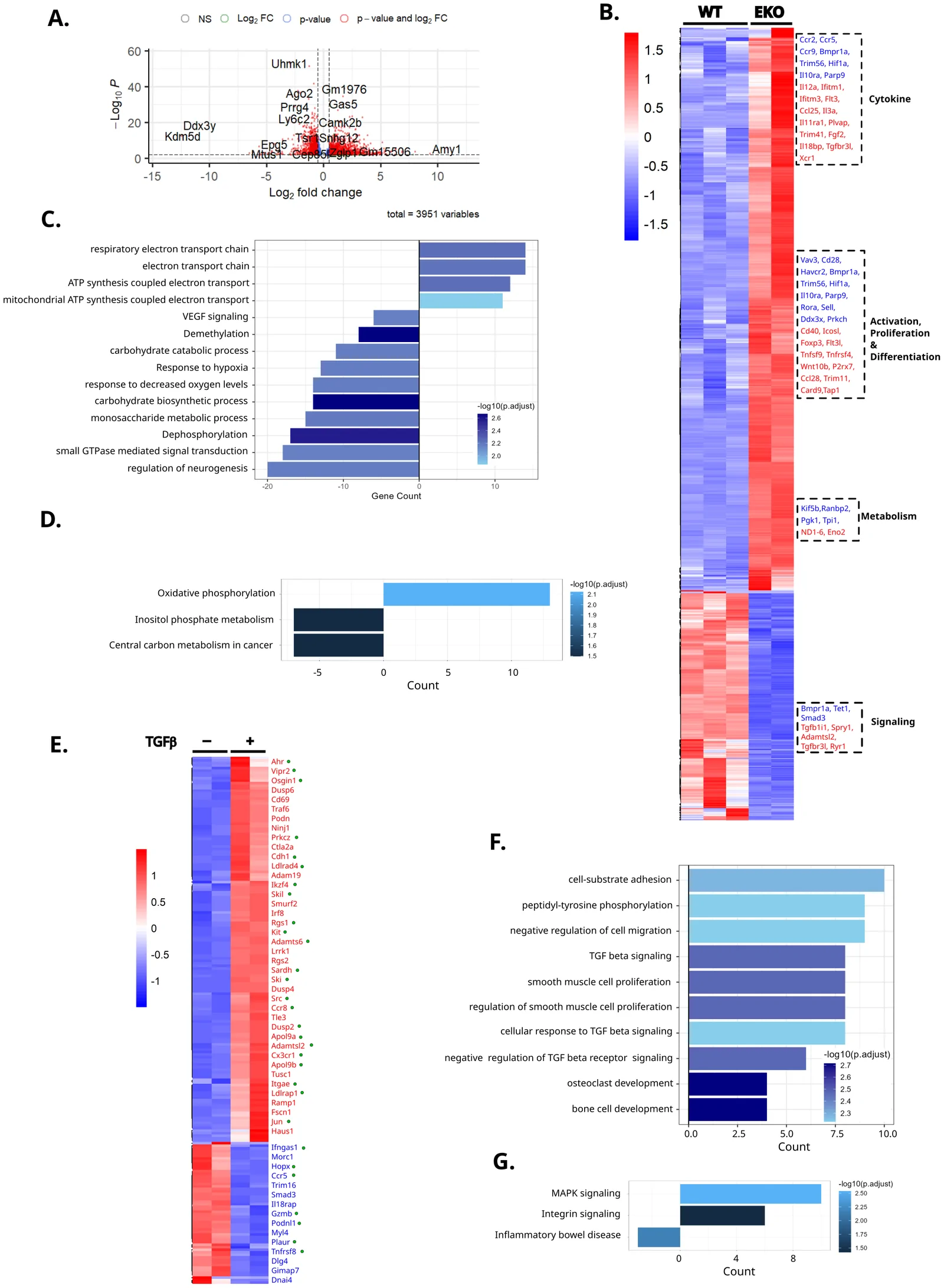
Loss of Eomes drives a transcriptional program similar to Smad4-deficient CD8 T cells. **(A)** Volcano plot and **(B)** Heatmap of differentially expressed genes in EomesKO CD8 T cells activated *in vitro* for 48 hours and expanded without TGFβ. **(C)** Gene Ontology and **(D)** KEGG analysis of differentially expressed genes. **(E)** Heatmap of differentially expressed genes in EomesKO CD8 T cells when exposed to TGFβ (10ng/ml). The green dot highlights the genes that are differentially expressed in WT CD8 T cells. **(F)** Gene Ontology and **(G)** KEGG analysis of differentially expressed genes in EomesKO CD8 T cells when exposed to TGFβ (10ng/ml). Each lane in the heatmap represents CD8 T cells isolated from one animal (WT (n=3), EomesKO (n=2) – 8–12 weeks old. All mice were females).

To determine whether the transcriptional programs regulated by Smad4 and Eomes are similar, we performed a similar RNA-seq analysis of Eomes-deficient CD8 T cells activated *in vitro* for 48 hours and then expanded further for an additional 12 hours in the presence or absence of TGFβ. In samples obtained immediately after 48 hours of activation, there were 6114 genes that were differentially expressed compared to WT, among which 2049 genes showed 2-fold or greater expression (**Supplementary Figures 2A, B**; **Supplementary Table 5**). Based on Gene Ontology and KEGG analysis, the differentially expressed genes were mostly involved in metabolic pathways and T cell differentiation similar to S4KO cells (**Supplementary Figures 2C, D**). When we looked at the gene expression profile of cells that were expanded for an additional 12 hours, we identified differential expression of 3951 genes (p_adj_ < 0.05) (**Figure 2A**) among which 1406 showed 2-fold or greater expression (**Figure 2B**; **Supplementary Table 6**). Loss of Eomes resulted in reduced expression of chemokine receptors involved in trafficking of effector cells (*Ccr2, Ccr5*, and *Ccr9*) (**Figure 2B**). Similar to S4KO, Eomes-deficient cells showed increased interferon-associated genes such as *Parp9, Ifitm1* and *Ifitm3* (**Figure 2B**). However, in contrast to S4KO cells, Eomes-deficient cells have reduced expression of genes associated with anti-inflammatory responses such as *Il10ra* and increased expression of cytokines (*Il12a*) and cytokine receptors, including *Il11ra1* and *Il3ra* (**Figure 2B**). Eomes-deficient CD8T cells mimicked S4KO cells in regulation of TCR signaling components and co-stimulatory molecules (**Figure 2B**). EomesKO cells have decreased expression of molecules involved in TCR signaling and activation (*Vav3, Prkch*), altered expression of co-stimulatory molecules [decreased *Cd28* and increased *Cd40, Icosl, Tnfsf9* (4-1BBL), *Tnfrsf4* (OX40)] and tissue homing molecules like *Sell* (L-selectin) and *Ccl28* (**Figure 2B**).

Eomes also exerted its control on genes involved in T cell metabolism, where Eomes-deficient cells have reduced expression of glycolytic genes (*Pgk1, Tpi1*, and *Hif1a*) and increased expression of *ND1-6*, which is similar to S4KO cells, suggesting increased reliance on oxidative phosphorylation (**Figure 2B**). Additionally, Gene Ontology and KEGG analyses revealed significant enrichment of genes involved in oxidative phosphorylation, mitochondrial respiration, and respiratory chain complex assembly (**Figures 2C, D**).

Compared to S4KO cells, TGFβ signaling was more active in EomesKO cells. We expanded EomesKO cells, 48 hours post-activation, in the presence of TGFβ for an additional 12 hours and subjected the cells to bulk RNA-seq analysis (**Figure 2E**; **Supplementary Table 7**). We identified 496 genes that were differentially expressed due to the addition of TGFβ, among which 208 genes showed 2-fold or greater expression (**Figure 2E**; **Supplementary Table 7**). GO and KEGG analysis confirm that TGFβ signaling is active in Eomes-deficient activated CTLs (**Figures 2F, G**). Interestingly, less than 50% of these genes were identical to the genes regulated by TGFβ in WT cells, indicating an altered TGFβ signaling in the absence of Eomes. Altogether, these findings indicate that Eomes is involved in similar transcriptional programs compared to Smad4 and that there is functional TGFβ signaling in the absence of Eomes.

### Smad4 and Eomes-deficient CD8 T cells share a dysregulated transcriptional program

**Figure 3:**
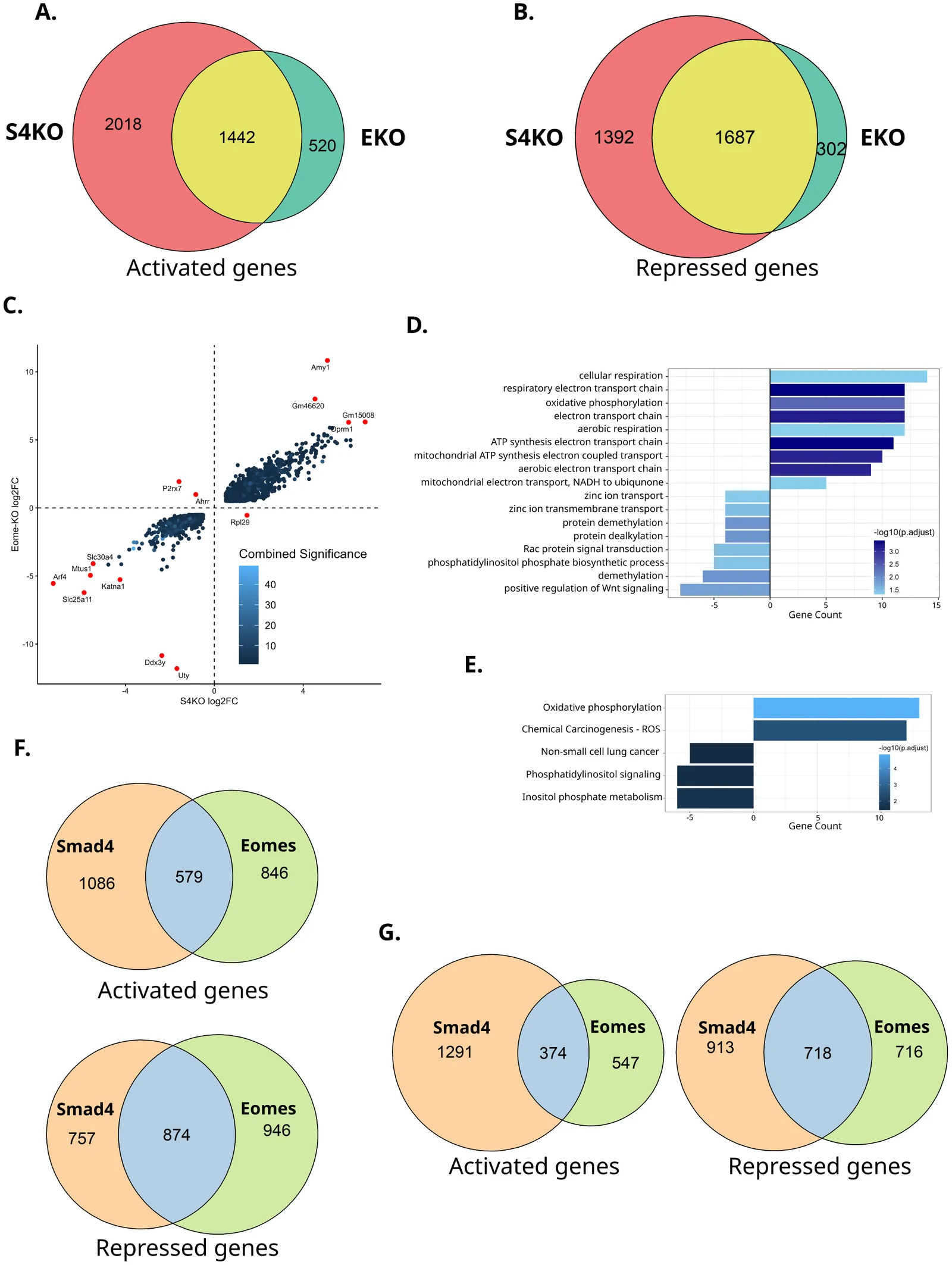
Smad4- and Eomes-deficient CD8 T cells share a dysregulated transcriptional program. **(A)** Venn diagram of activated and **(B)** repressed genes in S4KO and EomesKO CD8 T cells. **(C)** Scatter plot representing fold change of the shared set of genes between S4KO and EomesKO. **(D)** Gene Ontology and **(E)** KEGG analysis of the shared set of genes. **(F, G)** Venn Diagram of integrated RNAseq and ChIPseq analysis with putative direct target genes that were both differentially regulated (RNAseq) and bound by Smad4 and Eomes in promoter-proximal region genes in CD8 T cells with **(F)** high affinity TCR and **(G)** low affinity TCR.

To determine if the transcriptional program is shared by both Eomes and Smad4, we compared the differential gene expression of both datasets. Among the 6539 DEGs in S4KO and 3951 DEGs in EomesKO, 3129 were identically regulated in both gene sets in the same direction. We identified that 1442 genes were activated (**Figure 3A**) and 1687 genes were repressed (**Figure 3B**) in both Smad4- and Eomes-deficient cells. Among these genes, 866 have been expressed at 2-fold or more in the same direction, with only *P2rx7* being regulated in the opposite direction (**Figure 3C**).

Analysis of genes identically regulated by both Smad4 and Eomes revealed a shared transcriptional program characterized by reduced expression of genes involved in proximal TCR signaling [*Prkch, Pdpk1, Pkn1*, and *Inpp5d(SHIP1*)] and co-stimulation (*Cd28*) (**Supplementary Table 8**). Interestingly, it also led to the induction of survival and stress-associated genes such as *Lgals3* (Galectin-3), *Tnfsf*, and *Mdk* (**Supplementary Table 8**). Additionally, the expression of genes involved in the PI3K-Akt pathway (*Pik3cb, Pik3c2a, Pi4kb*, and *Pip4k2c*) is reduced, whereas genes involved in Wnt and Hedgehog signaling (*Wnt10b, Sfrp2, Lmbr1l*, and *Gli1*) are enriched in Smad4/Eomes-deficient cells. Based on these findings, we conclude that Eomes and Smad4 are associated with altered expression of genes involved in TCR-associated signaling pathways during differentiation towards the memory subset.

The absence of Smad4 or Eomes in activated CTLs altered the genes associated with glycolysis and metabolic regulation, where *Hif1a, Acly, Gbe1, Ppp1r3e*, and *Pdk1* were repressed (**Supplementary Table 8**) and genes involved in OXPHOS and mitochondrial electron transport chain (*ND1-6, COX1-3, Cytb*, and *Atp5f1e*) were increased (**Supplementary Table 8**). Accordingly, genes involved in mitochondrial homeostasis, autophagy, and stress responses (*Sirt4, Dguok, Slc25a27*, and *Atp6v1c2*) exhibited increased expression (**Supplementary Table 8**). This suggests that Smad4 and Eomes promote the expression of genes associated with glycolysis and prevent overactivation of mitochondrial respiration. Consistently, gene ontology analysis revealed marked enrichment for genes involved in mitochondrial bioenergetic processes such as cellular respiration, OXPHOS, mitochondrial respiratory chain complex assembly, and ATP biosynthetic process, suggesting that Smad4- and Eomes-deficient CD8 T cells have enhanced mitochondrial oxidative metabolism (**Figure 3D**). Similarly, KEGG analysis revealed enhanced enrichment of genes involved in OXPHOS and decreased expression of genes involved in the phosphatidylinositol signaling pathway and Inositol phosphate metabolism (**Figure 3E**).

To further investigate the role of Smad4 and Eomes, we integrated our RNAseq data with a previously published ChIPseq dataset for Smad4 and Eomes from activated CD8 T cells. This enabled the identification of putative direct target genes that were both differentially expressed (RNAseq) and bound by Smad4 and Eomes in the promoter proximal region (ChIPseq) (**Supplementary Table 9**). Notably, over 40% of genes directly activated by Eomes were also identified as direct Smad4 targets (**Figures 3F, G**), while approximately 50% of genes directly repressed by Eomes were similarly shared with Smad4 (**Figures 3F, G**). To independently verify these findings, we repeated the analysis using another published Eomes ChIPseq dataset and confirmed that a similar ratio of Eomes-regulated genes was shared with Smad4 target genes (**Supplementary Figure 3**). Contrastingly, analysis of a published ChIPseq dataset for the related T-box transcription factor T-bet identified only ~400 promoter-associated binding sites (**Supplementary Table 10**), compared with more than 2500 for Eomes. This suggests that extensive overlap between Smad4 and Eomes is specific to these two transcription factors.

### Abrogation of canonical TGFβ signaling defines a distinct and metabolically restrained state

**Figure 4:**
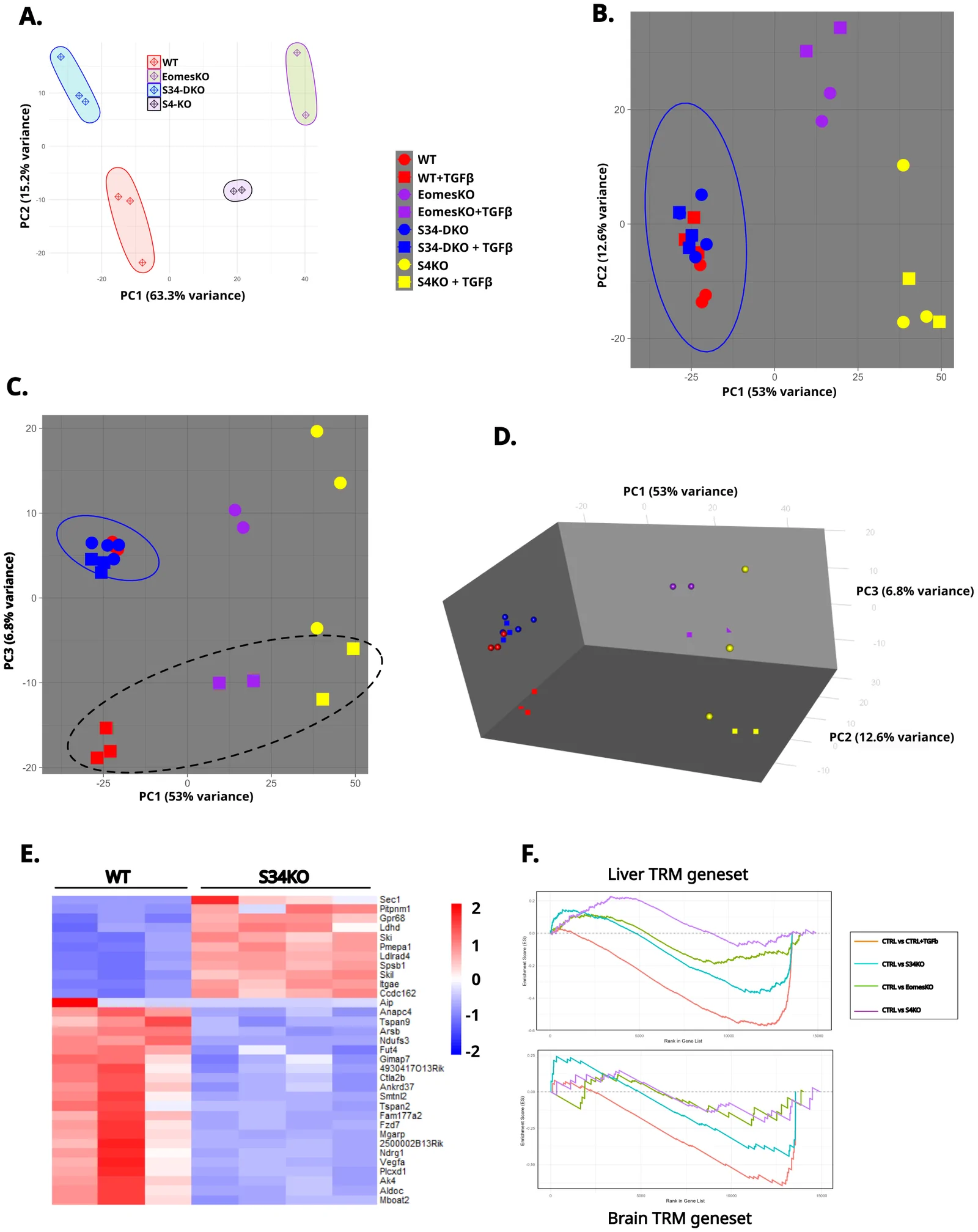
Abrogation of canonical TGFβ signaling defines a distinct and metabolically restrained state. **(A)** PCA plot of WT, EomesKO, S4KO, and S34-DKO after 48 hours of *in vitro* activation. **(B-D)** PCA plot of WT, EomesKO, S4KO, and S34-DKO after 48 hours of *in vitro* activation followed by expansion in the presence or absence of TGFβ. **(E)** Heatmap of differentially expressed genes in S34KO CD8 T cells activated *in vitro* for 48 hours and expanded without TGFβ. **(F)** GSEA plot comparing transcriptional profile of differentially expressed genes in S4KO, EomesKO, S34-DKO, and WT CD8 T cells treated with TGFβ against genes upregulated in liver T_RM_ cells (top) and brain T_RM_ cells (bottom). Each lane in the heatmap represents CD8 T cells isolated from one animal (WT (n=3), S34KOKO (n=4) – 8–12 weeks old. All mice were females.

The genes and metabolic pathways enriched in the absence of Smad4 and Eomes are consistent with the memory subset, and these cells also have dysregulated homing properties. Based on these findings, we investigated whether Smad4- and Eomes-deficiency biases the cells towards the resident memory subset. We used Smad3/Smad4 double knockout (S34-DKO) as a negative control with blockade of TGFβ signaling. Principal component analysis (PCA) of activated CD8 T cells revealed distinct clustering of samples according to genotype, with S4KO and S34-DKO cells relatively closer to the WT compared to EomesKO cells along the primary principal components (**Figure 4A**). Expansion of cells in the presence or absence of TGFβ for an additional 12 hours unraveled an unexpected phenotype where S34-DKO cells clustered together with WT cells along the primary principal components (**Figure 4B**). Using PC3, we observed a clearer separation across the genotype and TGFβ treatment (**Figure 4C**). S34-KO cells, irrespective of TGFβ treatment, clustered together with WT cells not exposed to TGFβ. On the contrary, WT, EomesKO, and S4KO cells treated with TGFβ clustered together, indicating an active signaling mechanism. Notably, in a 3D plot of PC1, PC2, and PC3, we observed Smad4- and Eomes-deficient cells clustered in close proximity, supporting a shared transcriptional program (**Figure 4D**).

Analysis of S34-DKO gene expression profile showed increased expression of genes associated with TGFβ negative feedback and signal attenuation (*Ski, Skil, Pmepa1, Ldlrad4*, and *Spsb1*) and increased expression of the tissue residency marker *itgae* (**Figure 4E**). In contrast to S4KO and EomesKO cells, the genes supporting mitochondrial function, OXPHOS, and stress adaptation were reduced. When these gene sets were compared with T_RM_ signature gene sets [Liver (36) and Brain (35)] using GSEA, we observed that WT cells treated with TGFβ as expected were enriched for TRM signature genes (p-value<0.05, FDR_q_value<0.05) (**Figure 4F**) (40). However, S34-DKO cells also expressed a TRM phenotype albeit at a decreased level compared to WT+ TGFβ (p-value<0.05, FDR_q_value<0.05) but at a higher level than S4KO and EomesKO (**Figure 4F**). These data indicate that combined loss of Smad3 and Smad4 favors a tissue-adaptive, metabolically restrained transcriptional program distinct from the metabolically amplified inflammatory state observed in EomesKO or S4KO CD8 T cells.

## Discussion

Viral infections and tumor cells trigger an immune response to clear the target cells, which is largely mediated by antigen-specific lymphocytes. However, when the target antigens are not eliminated, chronic exposure to antigens pushes the T cells towards a dysfunctional state. Hence, the type of viral infection and tumor has the potential to alter the fate of CD8 T cells. TGFβ signaling, which can be induced by viral infections and is a critical Epithelial-Mesenchymal Transition (EMT) component during cancer metastasis, plays a central role in the differentiation of activated CTLs (41). Naïve CD8 T cells are exposed to TGFβ in the context of migratory dendritic cells and preconditioned towards CD103+ T_RM_ (21). Hence, it is critical to prevent inappropriate diversion into memory or tissue-resident states when robust effector responses are required during early stages of an insult.

Smad4, an integral part of the TGFβ pathway, where it relays the signal from the extracellular ligand to the nucleus for gene regulation. Unlike TGFβR deficiency, which caused uncontrolled expansion of effector cells and increased expression of Ifnγ, perforin, and granzymes (22, 42), Smad4 deficiency did not have any effect on proliferation and caused impaired cytotoxicity (22, 27). Surprisingly, using different mouse models, multiple groups have unraveled a TGFβ−independent role for Smad4 during differentiation of CTLs (24, 26, 27). In particular, our group has shown that Smad4 inhibits the aberrant differentiation of T_RM_ and induces the differentiation of KLRG1+ T_EFF_ during the early stage of influenza infection. Similar to Smad4, Eomes has also been shown to restrict the differentiation of T_RM_ by directly suppressing the expression of *itgae*, the gene that codes for CD103 (5, 24, 26). Interestingly, the expression of Eomes is also controlled by TGFβ signaling through Smad4 (5, 24, 41).

In this study, we show that both Smad4 and Eomes share a transcriptional program that regulates genes involved in TCR signaling and metabolic programming during differentiation of activated CTLs. Using integrated RNAseq and ChIPseq analysis, we identified that Smad4 and Eomes regulate a significant number of genes similarly. This shared transcriptional program regulates the expression of genes that encode costimulatory molecules such as 4-1BBL and OX40L, which play a critical role in the survival of the activated cells (43). Furthermore, this includes genes involved in key developmental signaling pathways such as Wnt (44, 45), mTOR (46), and Hedgehog signaling (47), which have been implicated in lineage commitment, effector versus memory differentiation, and terminal exhaustion of CD8 T cells. In addition, it also regulates the genes encoding chemokines and chemokine receptors that are responsible for migration and homing in the peripheral tissues. This indicates that the shared transcriptional program between Smad4 and Eomes facilitates the differentiation and migration of activated CTLs, thereby enabling proper localization of CD8 T cells during an immune response.

Previous studies have demonstrated that Smad4 and Eomes are essential for memory cell differentiation (6, 22, 23, 48). The acquisition of memory cell fate is accompanied by extensive metabolic reprogramming, where CD8 T cells predominantly rely on mitochondrial OXPHOS to support their long-term survival and function (49). In contrast, we observed enrichment of genes involved in OXPHOS and mitochondrial respiration in Smad4- or Eomes-deficient CD8 T cells. These findings suggest that transcriptional regulation of genes involved in metabolic pathways alone is insufficient to establish a memory program. Future studies incorporating metabolic analysis will be required to determine whether these transcriptional changes result in altered mitochondrial metabolism.

Importantly, analysis of S34-DKO CD8 T cells revealed a transcriptional profile that was significantly different from that observed in S4KO cells. Unlike S4KO cells, which exhibited enrichment of OXPHOS-related genes, S34-DKO upregulated genes associated with tissue residency and negative regulators of TGFβ signaling. These findings are consistent with previous studies demonstrating that Smad4 restrains TRM differentiation while promoting T_EFF_ (23–27). Moreover, both our work and that of others have shown that TGFβ can promote T_RM_ differentiation in the absence of Smad4 (22, 24) but not in the absence of both Smad3 and Smad4. This indicates that canonical TGFβ signaling functions in the absence of Smad4. Together, these observations suggest that the canonical signaling mediated by the Smad3-Smad4 pathway and the shared transcriptional program by Smad4-Eomes exert non-redundant control over CD8 T cell fate.

Collectively, our findings identify that Smad4 and Eomes share a transcriptional program governing effector differentiation, metabolic reprogramming, and tissue residency in activated CD8 T cells. Rather than acting as a downstream mediator of the TGFβ pathway, Smad4 and Eomes appear to integrate multiple differentiation-associated networks to balance T_EFF_ and T_RM_ cell fates. This is critical particularly during a viral infection, where an effective immune response depends on generating sufficient effector cells while preventing aberrant commitment to T_RM_ fate as the naïve cells are preconditioned towards T_RM_ cells. Delineating the mechanisms behind such integrated transcriptional networks may help in distinguishing protective from pathogenic CD8 T cell responses during an infection, chronic inflammation, and cancer.

## Data Availability

The datasets presented in this study can be found in online repositories. The names of the repository/repositories and accession number(s) can be found in the article/**Supplementary Material**.

## Supplementary material

Supplementary Figure 1 Smad4 deficiency induces transcriptional reprogramming of genes involved in metabolism after 48 hours of activation. **(A)** Volcano plot and **(B)** Heatmap of differentially expressed genes in S4KO CD8 T cells activated *in vitro* for 48 hours. **(C)** Gene Ontology and **(D)** KEGG analysis of differentially expressed genes. Each lane in the heatmap represents CD8 T cells isolated from one animal (WT (n=3), S4KO (n=2) – 8–12 weeks old). Each lane in the heatmap represents CD8 T cells isolated from one animal (WT (n=3), EomesKO (n=2) – 8–12 weeks old). All mice were females.

Supplementary Figure 2 Loss of Eomes drives a transcriptional program similar to Smad4-deficient CD8 T cells after 48 hours of activation. **(A)** Volcano plot and **(B)** Heatmap of differentially expressed genes in EomesKO CD8 T cells activated *in vitro* for 48 hours. **(C)** Gene Ontology and D. KEGG analysis of differentially expressed genes. Each lane in the heatmap represents CD8 T cells isolated from one animal (WT (n=3), EomesKO (n=2) – 8–12 weeks old). Each lane in the heatmap represents CD8 T cells isolated from one animal (WT (n=3), EomesKO (n=2) – 8–12 weeks old). All mice were females.

Supplementary Figure 3 Smad4 and Eomes deficient CD8 T cells share a dysregulated transcriptional program. Venn Diagram of integrated RNAseq and ChIPseq analysis with putative direct target genes that were both differentially regulated (RNAseq) and bound by Smad4 and Eomes in promoter-proximal region genes in CD8 T cells.

Supplementary Table 1 Differential expression of genes in Smad4KO CD8 T cells after 48 hours of activation.

Supplementary Table 2 Differential expression of genes in Smad4KO CD8 T cells after 48 hours of activation with further expansion for 12 hours.

Supplementary Table 3 Differential expression of genes in wild-type CD8 T cells after 48 hours of activation with further expansion in the presence or absence of TGFβ for 12 hours.

Supplementary Table 4 Differential expression of genes in Smad4KO CD8 T cells after 48 hours of activation with further expansion in the presence or absence of TGFβ for 12 hours.

Supplementary Table 5 Differential expression of genes in EomesKO CD8 T cells after 48 hours of activation.

Supplementary Table 6 Differential expression of genes in EomesKO CD8 T cells after 48 hours of activation with further expansion for 12 hours.

Supplementary Table 7 Differential expression of genes in EomesKO CD8 T cells after 48 hours of activation with further expansion in the presence or absence of TGFβ for 12 hours.

Supplementary Table 8 Differential expression of genes that are shared between Smad4KO and EomesKO CD8 T cells after 48 hours of activation with further expansion for 12 hours.

Supplementary Table 9 Differential expression of genes that are shared between Smad4KO and EomesKO CD8 T cells after 48 hours of activation with further expansion for 12 hours and are bound by both Smad4 and Eomes in the promoter-proximal region.

Supplementary Table 10 Regions bound by Tbet in activated wild-type CD8 T cells.
